# Serum Magnesium, Prescribed Magnesium Replacement and Cardiovascular Events in Adults with Type 2 Diabetes: A National Cohort Study in U.S. Veterans

**DOI:** 10.3390/nu17132067

**Published:** 2025-06-21

**Authors:** Ying Yin, Yan Cheng, Andrew R. Zullo, Yijun Shao, Helen M. Sheriff, Charles Faselis, Simin Liu, Ali Ahmed, Qing Zeng-Treitler, Wen-Chih Wu

**Affiliations:** 1 Department of Clinical Research and Leadership, George Washington University, Washington, DC 20052, USA; yiny@email.gwu.edu (Y.Y.); yan_cheng@email.gwu.edu (Y.C.); yshao@email.gwu.edu (Y.S.); helen.sheriff@va.gov (H.M.S.); charles.faselis@va.gov (C.F.); ali.ahmed@va.gov (A.A.); zengq@email.gwu.edu (Q.Z.-T.); 2 Department of Medicine, Veterans Affairs Medical Center, Washington, DC 20420, USA; 3 Department of Medicine, Veterans Affairs Medical Center, Providence, RI 02908, USA; andrew_zullo@brown.edu; 4 Department of Epidemiology, School of Public Health, Brown University, Providence, RI 02912, USA; 5 Department of Medicine, Uniformed Services University, Washington, DC 20814, USA; 6 Department of Epidemiology & Biostatistics, Joe C. Wen School of Population and Public Health, University of California, Irvine, CA 92697, USA; simil10@hs.uci.edu; 7 Department of Medicine, Georgetown University, Washington, DC 20057, USA; 8 Department of Medicine, Alpert Medical School of Brown University, Providence, RI 02912, USA

**Keywords:** hypomagnesemia, hypermagnesemia, prescribed magnesium, magnesium replacement, cardiovascular disease, type 2 diabetes, MACE

## Abstract

Objectives: To investigate the relationship between serum magnesium levels, prescribed oral magnesium replacement, and major adverse cardiovascular events (MACE) in type-2 diabetes mellitus (T2D). Research design and methods: This nationwide retrospective study analyzed 1,284,940 US Veterans (≥18 years) with T2D who had outpatient serum magnesium testing between 1999–2021 in the Veterans Health Administration. The relationship between serum magnesium levels and MACE (hospitalizations for acute myocardial infarction, heart failure, ischemic stroke, or all-cause mortality) was determined using multivariable-adjusted Cox-regression models. Using a new-user-design and propensity-score-matching approach, we further related the use of prescribed oral magnesium and MACE among patients with hypomagnesemia (serum magnesium <1.8 mg/dL) and normomagnesemia (serum magnesium 1.8–2.3 mg/dL). Results: Of 1,284,940 patients with T2D, 229,210 (17.8%) patients had hypomagnesemia, and 117,674 (9.2%) patients had hypermagnesemia (serum magnesium >2.3 mg/dL). Compared to patients with normomagnesemia (serum magnesium 1.8–2.3 mg/dL), those with either hypomagnesemia or hypermagnesemia had elevated hazards for MACE. The risk increased with the severity of serum magnesium abnormalities in both directions—low (hazard ratios [HRs] 1.11–1.20) and high (HRs 1.04–1.39)—in a parabolic pattern. Oral magnesium was prescribed to 9.7% and 0.7% of patients with hypomagnesemia and normomagnesemia, respectively. After propensity-score-matching balanced across 64 baseline characteristics, oral magnesium was associated with a lower MACE risk in 40,766 matched patients with hypomagnesemia (HR 0.89; 95% confidence interval [CI], 0.84–0.93), especially those on proton-pump-inhibitors or thiazides. Oral magnesium was not related to MACE in 11,838 matched patients with normomagnesemia (HR 1.07; 95% CI, 0.97–1.17). Conclusions: In patients with T2D, both hypomagnesemia and hypermagnesemia were associated with higher one-year MACE risks compared to normomagnesemia. Prescribed oral magnesium was associated with a reduced MACE risk in hypomagnesemia but not in normomagnesemia.

## 1. Introduction

Magnesium is an intracellular mineral that is an integral part of more than 300 cellular enzymatic systems in the human body [1]. Primarily stored in bones and soft tissues, the intracellular concentration of magnesium is tightly controlled, and deviations from optimal levels, whether deficiency or excess, can lead to significant clinical implications. Although less than 1% of total magnesium circulates in the bloodstream, total serum magnesium measurements offer a practical estimation of overall magnesium reserves, particularly in clinical scenarios associated with magnesium deficiency.

Normal serum levels range from 1.8–2.3 mg/dL, and approximately 2% of the general population in the Western world experience hypomagnesemia. Individuals with type 2 diabetes experience approximately a tenfold higher rate of hypomagnesemia than that in the general population [2]. The interplay between magnesium levels in the human body and the pathogenesis of type 2 diabetes is bidirectional. Intracellular magnesium balance is crucial for maintaining peripheral glucose utilization [3], insulin signaling [4], and insulin secretion in pancreatic β cells. Low intracellular magnesium concentration is a direct factor in the development of insulin resistance, which may lead to type 2 diabetes [5]. Conversely, insulin also plays a significant role in regulating magnesium homeostasis by shifting extracellular magnesium into the cells [6]. Additionally, commonly prescribed medications used in diabetic patients, such as thiazide diuretics and proton pump inhibitors (PPIs), may exacerbate magnesium loss in the kidneys and gastrointestinal tract, respectively [7].

Cardiovascular disease (CVD) is the leading cause of death among patients with type 2 diabetes. Hypomagnesemia is linked to oxidative stress [8], inflammation [9], endothelial dysfunction, and atherosclerosis [10], further increasing the CVD risk in these patients. Studies have not been conclusive on the relationship between serum magnesium levels and major adverse cardiovascular events (MACE: acute myocardial infarction (AMI) [1], hospitalized heart failure (HF) [11], ischemic stroke, and all-cause mortality) in patients with type 2 diabetes, partly due to limited sample size [12,13].

Sufficient magnesium intake is essential for maintaining magnesium levels in the body. While magnesium-rich sources like whole grains, green leafy vegetables, beans, and nuts abound in natural food supplies, magnesium intake deficiency is common among adult Americans due to the increased consumption of processed foods [14]. Therefore, relying solely on the diet or supplements alone may be insufficient to maintain adequate body stores of magnesium, especially for patients with type 2 diabetes, who may need prescribed magnesium.

Meta-analyses of prospective cohort studies have indicated that increased dietary intake of magnesium-rich foods or the use of over-the-counter magnesium supplements is related to improvement in CVD risk factors [15,16,17] and risks of stroke [18,19] and HF [20,21]. However, there is a paucity of data relating prescribed oral magnesium in type 2 diabetes to MACE.

To address this gap, we leveraged the comprehensive electronic medical record (EMR) system from the Veterans Health Administration (VHA) and conducted a retrospective analysis of a nationwide cohort of Veterans with type 2 diabetes and serum magnesium levels. This study assesses the relationship between serum magnesium levels, prescribed oral magnesium for replacement, and MACE. We hypothesized that low serum magnesium levels are linked to adverse MACE outcomes, and prescribed oral magnesium may benefit those with hypomagnesemia.

## 2. Materials and Methods

### 2.1. Study Design

We conducted a retrospective study of a nationwide cohort of Veterans ≥18 years old diagnosed with type 2 diabetes using cross-linked EMR data from 1 January 1999 to 31 December 2022, within the VHA’s Corporate Data Warehouse (CDW), which includes comprehensive data on hospitalizations, clinic visits, laboratory results, and medications, and the VA-Medicare datasets to assess for healthcare utilization outside of the VHA for Medicare-eligible Veterans. We first evaluated the relationship between serum magnesium levels and MACE outcomes. We then examined the association between prescribed magnesium and MACE using a new-user design in patients with normal or low serum magnesium levels.

### 2.2. Eligibility Criteria

Patients with type 2 diabetes were identified by two type 2 diabetes International Classification of Diseases (ICD) codes (Table S1), one ICD code and one type 2 diabetes medication (Table S2), or one ICD code and hemoglobin A1c ≥6.5%, based on the VHA’s “Diabetes Epidemiology Cohorts” criteria [22]. We identified 3,448,558 Veterans who had a new diagnosis of type 2 diabetes and received care within VHA between 1999 and 2021. We excluded 14 patients who were <18 years of age, 978,176 patients without at least a one-year history of VHA care, and 1,185,428 patients without a serum magnesium test for a final study sample of 1,284,940 patients to relate serum magnesium levels to MACE. Of these patients, 229,210 patients had hypomagnesemia (serum magnesium < 1.8 mg/dL), 938,056 patients had normomagnesemia (serum magnesium 1.8–2.3 mg/dL), and 117,674 patients had hypermagnesemia (serum magnesium > 2.3 mg/dL). Henceforward, the date of the ambulatory magnesium test was used as the index date.

**Figure 1:**
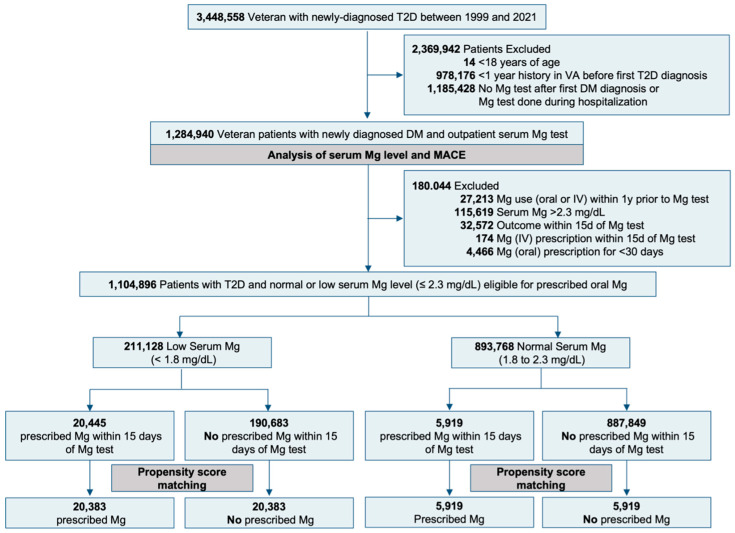
Assembly of the Study Cohort.

To relate prescribed oral magnesium and MACE, we excluded patients who received prescribed oral and intravenous magnesium in the year before serum magnesium test (*n* = 27,213) to avoid prevalent user bias, patients who had hypermagnesemia (serum magnesium > 2.3 mg/dL) as they would not be eligible to receive magnesium replacement (*n* = 115,619), and those who experienced MACE within 15 days of magnesium test (*n* = 32,572) to provide a 15-day blanking period allowing time for magnesium prescription (Figure 1). Thus, this analysis was restricted to patients with type 2 diabetes who were eligible for prescribed oral magnesium replacement therapy, including 211,128 patients with hypomagnesemia and 893,768 patients with normomagnesemia.

### 2.3. Treatment Strategies

Given that oral replenishment of the body’s magnesium stores takes time [23], patients from our initial cohort eligible for prescribed magnesium were allocated to the treatment group if they received a ≥30-day prescription for oral magnesium within 15 days following a serum magnesium test (*n* = 4466 excluded). Data on magnesium prescriptions (including magnesium oxide, magnesium gluconate, magnesium chloride, and magnesium sulfate) were obtained from VHA pharmacy data. Magnesium citrate and magnesium hydroxide were excluded, as they were primarily used for bowel regimens. As we are only interested in oral magnesium, patients with intravenous magnesium prescriptions within 15 days were also excluded (*n* = 174). All analyses focused on an intention-to-treat estimand—the effect of initiating magnesium versus not, regardless of subsequent treatment discontinuation or switching among treatment groups.

### 2.4. Outcomes

The primary outcome was MACE: hospitalizations with AMI, HF, ischemic stroke, or all-cause mortality within one year of the index date, with the last date of outcome accrual being 31 December 2022. Data on hospitalizations were abstracted using ICD codes (Table S1) from VHA hospital records and Medicare claims for non-VHA facilities. The secondary outcome was all-cause mortality. Patients’ vital status and death date were obtained from the VHA CDW, which compiled records from death certificates, the Social Security Administration Death Master File, and the Centers for Medicare & Medicaid Services Vital Status Files.

### 2.5. Covariates

Selected baseline conditions were defined using ICD codes (Table S1) documented at any time before the index date. Baseline Gagne comorbidity scores [24], a weighted score to assess overall comorbidity, were calculated. Medications (Table S2) were identified from outpatient pharmacy data within one year before the index date. We also gathered the most recent data for vital signs and laboratory values (Table S3) within one year prior to the index date. The calendar year of the index date, patients’ geographic location by Veterans-Integrated-Services-Networks (VISNs), and the rurality of the residence were included for a total of 64 covariates extracted (Figure S1).

Missing values were coded as not present for categorical variables. For continuous variables (Table S4), missing values were imputed by a single imputation with a fitted general linear model on age, sex, race, and ethnicity. Due to the large sample size, absolute standardized differences (ASD) were calculated to compare baseline covariates between the analyzed groups.

### 2.6. Statistical Analysis

#### 2.6.1. Serum Magnesium Level and MACE Outcome

We performed multivariable Cox regression analysis adjusted for 64 baseline covariates to assess the association between serum magnesium levels and one-year MACE outcomes.

#### 2.6.2. Propensity Score Matching

To estimate the association between prescribed magnesium and outcomes, we used propensity scores (PS) to assemble balanced cohorts of prescribed magnesium versus no prescribed magnesium in an outcome-blinded manner, mimicking a randomized controlled trial (RCT) [25,26]. To analyze patients with hypomagnesemia, we used a non-parsimonious multivariable logistic regression model to calculate PS for the receipt of prescribed magnesium for each of the 211,128 patients based on the 64 baseline characteristics. We used a greedy matching protocol to match patients with and without prescribed magnesium based on PS to five, four, three, two, and one decimal places in five steps. This yielded 20,383 patients in each group, forming a matched cohort of 40,766 patients (Figure 1). The balance in baseline characteristics was measured using ASD in the post-match cohort [27]. An ASD < 10% is considered inconsequential. For patients with normomagnesemia, we repeated the above approach and assembled a PS-matched cohort of 11,838 patients with 1:1 matching (Figure 1).

The Kaplan–Meier method was used to estimate the cumulative probabilities of MACE and death. After confirming the assumption of risk proportionality by visualizing the Schoenfeld residuals, we used Cox regression models to estimate hazard ratios (HRs) and 95% confidence interval (95% CI) for the outcomes [28,29].

#### 2.6.3. Sensitivity Analysis

To assess the robustness of the effect estimates using an alternative analytic methodology, we also determined the association between prescribed magnesium and outcomes using multivariable Cox regression models adjusted for all 64 covariates in the pre-PS-matched cohort for the hypomagnesemia and the normomagnesemia groups, respectively, and estimated adjusted HRs (95% CI).

#### 2.6.4. Subgroup Analyses

Subgroup analyses were performed to evaluate whether the association between prescribed magnesium and outcomes varied across participant characteristics (i.e., effect measure modification). Subgroups were based on demographics (sex and race), serum magnesium levels, comorbidity (HF), medications that can lead to hypomagnesemia (diuretics, PPIs, metformin) or interacts with magnesium (vitamin D) to determine for heterogeneity of associations, if any, between prescribed magnesium and MACE, in the PS-matched cohort for both hypomagnesemia and normomagnesemia groups.

For each subgroup variable, we fit separate PS within each subgroup stratum, and individuals were re-matched to ensure a covariate balance between treatment groups within subgroups, confirmed using ASD < 0.10 [30]. After PS-matching, Cox proportional hazards regression was employed to estimate the treatment effects on MACE outcome within each subgroup. We then aggregated data from the propensity-score-balanced strata into a single dataset and fit regression models, including interaction terms between prescribed magnesium and each subgroup variable (i.e., each in a separate model), to estimate *p*-values for homogeneity. A *p*-value below 0.05 indicated a statistically significant difference in the estimated effect across subgroup strata and potential effect modification by that subgroup variable.

#### 2.6.5. Software

Statistical analyses were conducted using Python 3.8.3. The graphics were generated using the Lifeline 0.27.7, scikit-learn 1.2.2, SciPy 1.8.0, and Matplotlib 3.2.2 packages.

## 3. Results

### 3.1. Serum Magnesium and MACE in Type-2 Diabetes

Of the 1,284,940 patients with ambulatory serum magnesium levels after type 2 diabetes diagnosis, 229,210 (17.8%) patients had hypomagnesemia, and 117,674 (9.2%) patients had hypermagnesemia. Both hyper- and hypomagnesemia patients had a higher comorbidity burden measured by Gagne’s comorbidity score compared to patients with normomagnesemia. Patients with hypomagnesemia had a longer diabetes duration and a higher prevalence of hypertension, anemia, alcohol abuse, liver disease, and metformin use. Conversely, hypermagnesemia patients were more likely to have a history of HF, respiratory failure, or reduced kidney function (Table 1).

**Figure 2:**
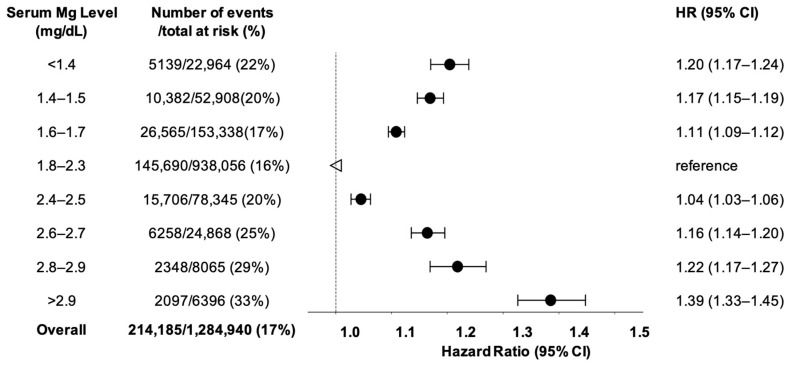
Adjusted Hazard Ratios for Baseline Magnesium Levels and Time to MACE in Patients with Type 2 Diabetes. The arrow indicates the normal serum magnesium level, which serves as the reference. The dots with error bars represent the adjusted hazard ratios (95% confidence intervals) for time to MACE within one year of an ambulatory magnesium test in patients with type 2 diabetes and varying serum magnesium levels, compared with patients with type 2 diabetes and normal serum magnesium levels. The analysis used a multivariate Cox regression model adjusted for 64 variables, including demographics, comorbidities, medication history, and recent lab tests. Abbreviation: CI = Confidence Interval; HR = Hazard Ratio; MACE = major adverse cardiac events.

Compared to patients with normomagnesemia, patients in either hypomagnesemia or hypermagnesemia groups were associated with elevated hazards for MACE. The hazards increased with the severity of the serum magnesium abnormality in both high and low magnesium levels in a parabolic pattern. Specifically, patients with serum magnesium levels <1.4 mg/dL had a 20% higher risk, and those with levels >2.9 mg/dL had a 39% higher risk of MACE at one year, compared with the normomagnesemia group (Figure 2).

### 3.2. Prescribed Oral Magnesium and Outcomes

Prescribed oral magnesium was observed in 9.7% of patients with hypomagnesemia and 0.7% of patients with normomagnesemia. The median duration of magnesium prescription was 90 days (interquartile range [31]: 30–176 days), with a median dosage of 420 mg/day (range: 140–500 mg/day). Approximately 99% of the prescriptions were for magnesium oxide.

#### 3.2.1. Oral Magnesium in Hypomagnesemia

Compared to patients without magnesium prescription, patients with prescribed magnesium were more likely to be of White race, have a longer diabetes duration, and have a higher prevalence of hyperlipidemia, hypertension, and treatment with metformin, PPIs, vitamin D, and statins (Table 2).

After PS-matching, *n* = 20,383 patients with prescribed magnesium were PS-matched to *n* = 20,383 patients without magnesium prescription with adequate balance on 64 baseline covariates (Figure S1A). The patients in the PS-matched cohort had a mean (SD) age of 66 (10) years and were comprised of 5% women and 72% White, with a mean (SD) serum magnesium level of 1.5 (0.2) mg/dL and an estimated glomerular filtration rate (eGFR) of 74.1 (22.5) mL/min/1.73 m^2^.

**Figure 3:**
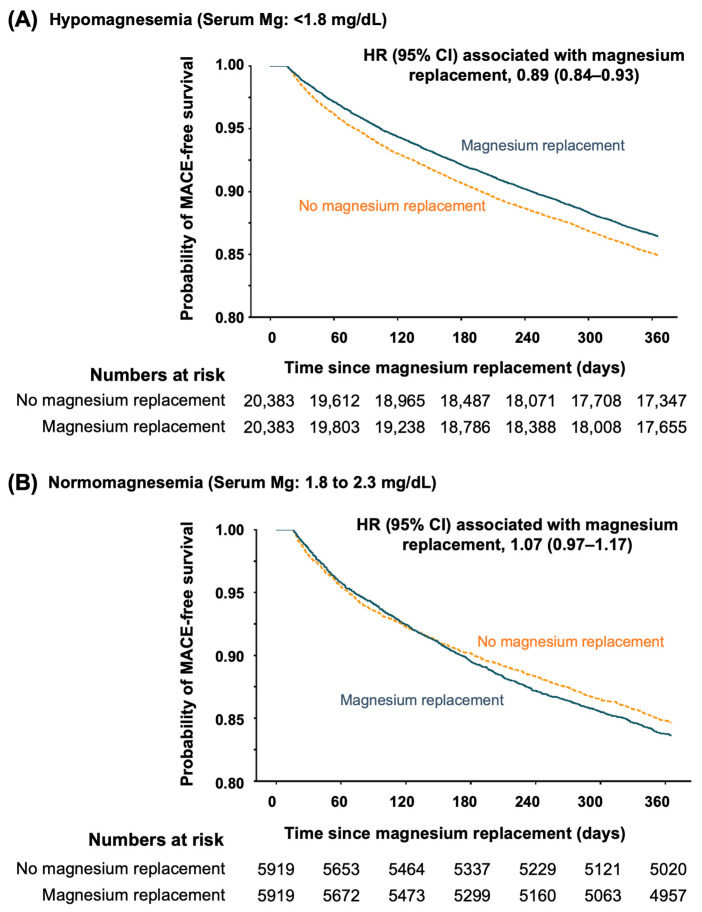
One-Year Kaplan–Meier Curves for MACE-Free Survival by Initiation of Prescribed Magnesium in Propensity Score-Matched Cohorts of Patients with Type 2 Diabetes and (**A**) Hypomagnesemia, or (**B**) Normomagnesemia. Abbreviation: MACE = major adverse cardiovascular events.

In these PS-matched patients, MACE occurred in 13.6% of the prescribed magnesium and 15.1% of the non-magnesium groups (Table 3). Cox-regression showed that prescribed magnesium was associated with decreased one-year risks of MACE, HR 0.89 (95%CI 0.84–0.93) (Figure 3A) and death (HR: 0.86 (95% CI, 0.80–0.93) (Figure S2A). Sensitivity analyses using multivariate Cox regression modeling applied to the pre-PS-matched cohort of patients yielded similar results. The adjusted HRs associated with prescribed magnesium compared to non-magnesium were 0.93 (95% CI, 0.89–0.96) for MACE and 0.93 (95% CI, 0.88–0.99) for all-cause mortality (Table 3).

**Figure 4:**
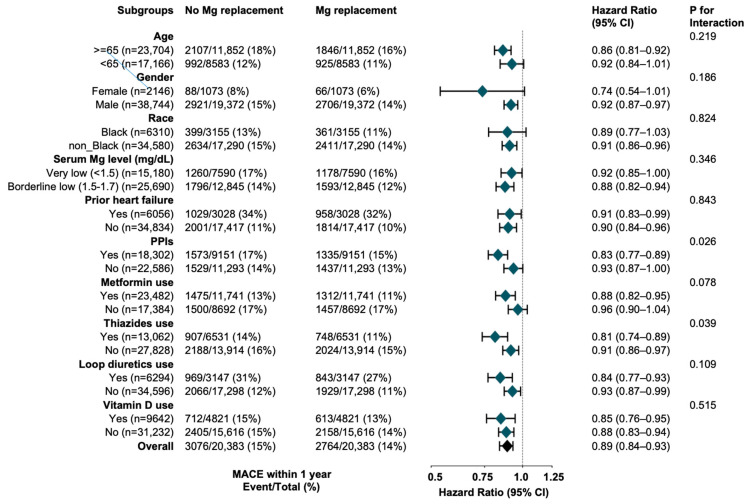
Hazard Ratios for the Association of Prescribed Magnesium and Time to MACE Within One Year in Subgroup-Specific Propensity-Matched Cohorts of Patients With Type 2 Diabetes and Hypomagnesemia. Abbreviation: CI = Confidence Interval; HR = Hazard Ratio; MACE = major adverse cardiovascular events; PPIs = Proton Pump Inhibitors.

The association between prescribed magnesium and MACE in the PS-matched cohort was homogeneous across subgroups of age, sex, race, serum magnesium, HF, and certain medications (metformin, loop diuretics, and vitamin D). Exceptions were noted for subgroups stratified by the use of PPIs (*p* = 0.026 for interaction) or thiazides (*p* = 0.039 for interaction), where the association of prescribed magnesium with lower risks of MACE was more substantial for those on PPIs or thiazides compared to those not on the respective medication(s) (Figure 4).

#### 3.2.2. Oral Magnesium in Normomagnesemia

Compared to patients without magnesium prescription, those in the prescribed magnesium group had a higher Gagne comorbidity score and a higher prevalence of a history of HF, atrial fibrillation, anemia, alcohol abuse, liver disease, and neurologic disorders. They were also more likely to be treated with loop diuretics, non-selective beta-blockers, mineralocorticoid-receptor-antagonists, and PPIs (Table S5).

After PS-matching, *n* = 5919 patients with prescribed magnesium were PS-matched to *n* = 5919 patients without magnesium prescription (Figure S1B). The patients in the PS-matched cohort had an average (SD) age of 65 (11) years, were comprised of 6% women and 68% White, and had a mean (SD) serum magnesium level of 2.0 (0.2) mg/d and an eGFR of 73.9 (24.5) mL/min/1.73 m^2^.

In the PS-matched cohort of the normomagnesemic group, MACE occurred in 16.4% of patients who received prescribed magnesium compared to 15.4% in the non-magnesium group (Table 3). Prescribed magnesium was not significantly associated with risks of MACE [HR 1.07 (95% CI, 0.97–1.17)] (Figure 3B), or death [HR 1.00 (95% CI, 0.87–1.15)] (Figure S2B). Subgroup analyses showed the association between prescribed magnesium and MACE in the normomagnesemic PS-matched cohort to be homogeneous, except for subgroups stratified by the use of PPIs (*p* = 0.007 for interaction), which showed a higher risk of MACE in patients with prescribed magnesium and not on PPIs (Figure S3).

Sensitivity analyses using multivariate-Cox-regression modeling in the pre-PS-matched cohort showed that prescribed magnesium in normomagnesemic patients was associated with higher risks of MACE, HR 1.14 (95% CI 1.07–1.21), and death, HR 1.11 (95% CI 1.01–1.23).

## 4. Discussion

In a nationwide cohort of over 1.2 million Veterans with type 2 diabetes, 18% had hypomagnesemia, and 9% had hypermagnesemia. Both hypomagnesemia and hypermagnesemia were associated with higher risks of one-year MACE and death compared to patients with normomagnesemia. In patients with hypomagnesemia, prescribed magnesium was associated with reduced one-year risks of MACE and death, whereas in patients with normomagnesemia, prescribed magnesium was not associated with MACE.

### 4.1. Hypomagnesemia and Cardiovascular Outcomes

Hypomagnesemia is notably prevalent among individuals with type 2 diabetes, with previous studies reporting a prevalence ranging from 13.5% to 47.7% [2]. In our study, we observed a similar prevalence of hypomagnesemia (18%) within the VHA cohort of patients with type 2 diabetes. Our data suggest that patients with longer diabetes duration and those undergoing treatment with insulin, metformin, or PPIs are at higher risk of developing hypomagnesemia.

Previous research on the relationship between low serum magnesium levels and increased mortality has focused on critically ill inpatients or those with end-stage renal disease [32,33,34,35,36]. Observations from outpatient cohorts with type 2 diabetes have been inconsistent in relating serum magnesium levels to MACE, likely due to limited sample sizes. A cross-sectional study (*n* = 450) of patients with type 2 diabetes demonstrated a lower incidence of coronary heart disease with higher serum magnesium levels [37]. In contrast, the Fremantle Diabetes Study (*n* = 940) identified an inverse association between serum magnesium and stroke but not coronary heart disease [13]. Another study of 4348 patients with type 2 diabetes found an inverse relationship between serum magnesium and incident HF and atrial fibrillation, but not with MACE [12]. Our nationwide ambulatory cohort of 1.2 million patients with type 2 diabetes builds upon these results by not only showing the associated risks of hypomagnesemia with MACE and death but also the association with improved outcomes with prescribed magnesium.

Potential mechanisms that may relate low magnesium to MACE and death in patients with type 2 diabetes may be due to the worsening of several CVD risk factors [15,16,17]. Magnesium deficiency promotes insulin resistance [38], worsens glycemic control [39], and is associated with hypertension and chronic inflammation. Additionally, it increases oxidative stress [8] and endothelial dysfunction, accelerates atherosclerosis [10], and disrupts the renin-angiotensin-aldosterone system (RAAS), leading to cardiac fibrosis and hypertrophy, all of which may increase the risk of thromboembolic events such as AMI or stroke and worsening of HF [40].

Magnesium supplementation has been shown to improve insulin resistance, hyperglycemia [41,42], inflammation, endothelial dysfunction, and hypertension [43] in trials of 4–26 weeks. Research also demonstrated that even short-term (<3 weeks) Magnesium supplementation can reduce the risk of ventricular arrhythmias in heart failure [44,45,46]. In our study, the median duration of magnesium prescription was 90 days (interquartile range [31], 30–176 days), making it biologically plausible that prescribed magnesium corrects hypomagnesemia, stabilizes cardiac function, and improves cardiovascular outcomes.

### 4.2. Hypermagnesemia and Cardiovascular Outcomes

In our cohort, we observed a prevalence of approximately 9% for hypermagnesemia. The risk of hypermagnesemia is particularly elevated in individuals with decreased kidney function, indicated by lower eGFR values.

Previous studies have suggested an association between hypermagnesemia and adverse cardiovascular outcomes. For example, a meta-analysis [11] reported an elevated risk of CVD mortality among patients with HF who had higher serum magnesium levels. Additionally, an increase in in-hospital mortality and worse clinical outcomes were observed in hospitalized patients with hypermagnesemia in several other studies [31,47,48]. A recent cross-sectional study (*n* = 316) also found that hypermagnesemia was associated with a higher prevalence of microvascular complications in diabetes [49].

To our knowledge, our study is the first to demonstrate an association between hypermagnesemia and an increased one-year risk of MACE in an ambulatory population of patients with type 2 diabetes, using a large cohort of over 100,000 patients.

Potential mechanisms by which hypermagnesemia can cause harm include muscular weakness (including respiratory muscles) and discoordination, profound fatigue, hypotension, bradycardia, and other cardiac dysrhythmias, as well as confusion and altered mental status [50].

### 4.3. Clinical Implications

These findings have significant clinical implications. Monitoring serum magnesium levels is currently not a standard practice in the outpatient management of patients with type 2 diabetes. Our results suggest that this should be considered in certain patients at risk, as both low and high magnesium levels were associated with adverse outcomes. Examples of such patients from our results may include those with cardiac or neurologic disorders, alcohol abuse, liver disease, abnormal kidney function, and those on medications that may alter magnesium excretion, such as thiazide diuretics, PPIs, or metformin. The subgroup analyses of patients with hypomagnesemia indicated that the impact of prescribed magnesium was more pronounced in patients taking PPIs or thiazides. These findings also highlight opportunities for practice improvement since prescribed magnesium was observed in <10% of patients with hypomagnesemia. The finding that prescribed magnesium is not associated with improved outcomes in patients with normomagnesemia is novel. It suggests that the body’s magnesium levels are maintained within a narrow normal range for optimal function [1,6,51].

### 4.4. Limitations

This study has several limitations. First, the study population was predominantly male, which may limit the generalizability of our findings to females. However, our cohort included 11,705 female patients in the hypomagnesemia group and 44,640 in the normomagnesemia group, substantial numbers compared with other studies. Second, while we combined VHA and Medicare data for outcome ascertainment, patients seeking care from other payers may have been missed, leading to potential misclassification bias and underestimating the associations. Third, restricting the sample to patients with serum magnesium levels may introduce a potential selection bias, possibly favoring the inclusion of a higher-risk population with comorbidities or conditions necessitating magnesium testing; as such, our results should be interpreted accordingly. However, it is important to note that our study cohort represents over half of the VHA users with type 2 diabetes, suggesting that we captured a substantial proportion of the at-risk population. Fourth, despite our new-user design, propensity-matching, sensitivity, and subgroup analyses to minimize confounding, residual confounding may still exist, given the study’s observational nature. This limitation can be overcome by conducting a large-scale RCT in the future. Fifth, our replacement study was limited by evaluating a binary treatment strategy (magnesium replacement vs. no replacement), without accounting for the potential effects of varying magnesium dosages and treatment durations. Future research should explore these dose-response relationships and with extended follow-up periods, which would provide a clearer understanding of the long-term associations between serum magnesium levels, supplementation, and MACE. Furthermore, we did not conduct a follow-up analysis to examine changes in serum magnesium levels over time, which limits our ability to assess the long-term impact of magnesium replacement therapy on both serum magnesium levels and cardiovascular risk.

## 5. Conclusions

In summary, our study showed that in patients with type 2 diabetes, both hypomagnesemia and hypermagnesemia were associated with higher one-year risks of MACE and death compared to normomagnesemia. Prescribed oral magnesium was associated with reduced risks of MACE and death in hypomagnesemia but not in normomagnesemia.

## Figures and Tables

**Table 1:** Baseline Characteristics of Patients with Type 2 Diabetes Categorized by Three Serum Magnesium Levels.

	Serum Magnesium Level, mg/dL		
Characteristic, n (%)	Hypomagnesemia (<1.8)	Normomagnesemia (1.8 to 2.3)	Hypermagnesemia (>2.3)
	(*n* = 229,210)	(*n* = 938,056)	(*n* = 117,674)
Age, Mean (SD), y	65.7 (10.9)	65.6 (11.8)	67.0 (12.0)
Sex			
Male	217,505 (95)	893,416 (95)	113,747 (97)
Female	11,705 (5)	44,640 (5)	3927 (3)
Diabetes Duration, Mean (SD), y	4.6 (4.5) ^e^	3.6 (4.0)	2.9 (3.6) ^e^
Serum Magnesium, Mean (SD), mg/dL	1.6 (0.2) ^e^	2.0 (0.2)	2.6 (0.3)
Race			
Hispanic	12,198 (5)	55,066 (6)	5750 (5)
non-Hispanic White	154,197 (67)	608,270 (65)	75,214 (64)
non-Hispanic Black	41,876 (18)	179,829 (19)	23,179 (20)
Other/Unknown	20,939 (9)	94,891 (10)	13,531 (11)
Marital Status			
Single	22,028 (10)	91,298 (10)	11,332 (10)
Divorced/Separated	66,173 (29)	258,158 (28)	31,781 (27)
Married	121,614 (53)	506,738 (54)	61,898 (53)
Widowed	19,395 (8)	81,862 (9)	12,663 (11)
Cormobidities ^a^			
Heart Failure	35,282 (15)	143,957 (15)	25,978 (22) ^e^
AMI	26,147 (11)	107,176 (11)	16,347 (14)
Ischemic stroke	22,202 (10)	88,633 (9)	12,939 (11)
Hemorrhagic stroke	2126 (1)	8184 (1)	1123 (1)
Atrial Fibrillation	30,526 (13)	117,157 (12)	18,580 (16)
Hyperlipidemia	183,775 (80)	745,272 (79)	92,170 (78)
Hypertension	205,182 (90) ^e^	803,045 (86)	101,232 (86)
Anemia	67,440 (29) ^e^	220,724 (24)	32,201 (27)
Alcohol abuse	50,968 (22)^e^	170,155 (18)	20,419 (17)
Liver Disease	34,260 (15) ^e^	100,288 (11)	10,710 (9)
Respiratory Failure	10,803 (5)	35,305 (4)	7128 (6) ^e^
COPD	62,578 (27)	244,926 (26)	34,745 (30)
Cancer	75,523 (33)	287,773 (31)	36,914 (31)
Neuro Disorders	82,647 (36)	333,242 (36)	36,471 (31)
Weight Loss	20,619 (9)	68,358 (7)	10,111 (9)
Arthritis	107,185 (47)	436,185 (46)	52,159 (44)
Gagne Comorbidity Score ^b^	2.3 (2.5) ^e^	2.0 (2.4)	2.4 (2.6) ^e^
Comedications ^c^			
Diabetes Medications			
Insulin	45,470 (20) ^e^	115,937 (12)	12,233 (10)
Metformin	108,915 (48) ^e^	298,678 (32)	23,799 (20) ^e^
GLP1	2818 (1)	6136 (1)	405 (0)
SGLT2 inhibitors	1857 (1)	9109 (1)	971 (1)
Other Diabetes Medication	67,608 (29) ^e^	196,048 (21)	22,621 (19)
Other Medications			
Thiazides	64,467 (28)	233,432 (25)	30,027 (26)
Loop Diuretics	31,269 (14)	131,606 (14)	25,321 (22) ^e^
PPIs	85,687 (37) ^e^	284,807 (30)	33,744 (29)
Vitamin D	42,932 (19)	151,372 (16)	14,697 (12) ^e^
ACEIs	110,178 (48) ^e^	398,710 (43)	51,173 (43)
ARBs	29,014 (13)	103,785 (11)	11,864 (10)
Other anti-hypertension	55,274 (24)	210,594 (22)	31,094 (26)
Selected beta blockers	76,746 (33)	276,890 (30)	39,030 (33)
Non-selected beta blockers	24,755 (11)	90,112 (10)	12,629 (11)
Digoxin or Other Inotropes	9124 (4)	40,934 (4)	7256 (6)
Aspirin	49,509 (22)	195,517 (21)	27,703 (24)
Anti-platelet	18,415 (8)	72,019 (8)	10,707 (9)
Glucocorticoids	24,240 (11)	93,739 (10)	12,834 (11)
Statins	136,168 (59)	525,964 (56)	66,209 (56)
Other non-statin lipid lowering	23,527 (10)	90,287 (10)	13,781 (12)
Calcium Channel Blocker	63,174 (28)	242,155 (26)	33,705 (29)
MRAs	9951 (4)	31,537 (3)	5490 (5)
Health examination data ^d^			
HbA1c, Mean (SD), %	7.4 (1.8) ^e^	7.0 (1.6)	6.9 (1.6) ^e^
BMI, Mean (SD), kg/m^2^	30.7 (6.8)	30.8 (6.6)	29.9 (6.4) ^e^
Systolic BP, Mean (SD), mmHg	134.5 (19.8)	134.6 (19.1)	132.4 (20.2) ^e^
Diastolic BP, Mean (SD), mmHg)	76.0 (11.8)	76.4 (11.8)	74.2 (12.5) ^e^
eGFR, Mean (SD), mL/min/1.73 m^2^	75.1 (24.3)	74.6 (23.4)	62.9 (27.1) ^e^
Serum Vitamin D, Mean (SD), ng/mL			
<20	14,650 (6)	57,252 (6)	5535 (5)
20–30	21,273 (9)	88,333 (9)	8251 (7)
30–100	35,754 (16)	134,419 (14)	12,611 (11) ^e^
Unknown	157,533 (69)	658,052 (70)	91,277 (78) ^e^
Serum Sodium, Mean (SD), mEq/L	137.7 (3.8) ^e^	138.4 (3.4)	138.6 (4.5)
Serum Potassium, Mean (SD), mEq/L	4.1 (0.5) ^e^	4.2 (0.5)	4.3 (0.6) ^e^
Serum Calcium, Mean (SD), mg/dL	9.1 (0.8) ^e^	9.2 (0.6)	9.1 (0.7)
LDL cholesterol, Mean (SD), mg/dL	86.4 (35.3) ^e^	94.9 (38.8)	97.0 (37.0)
Triglycerides, Mean (SD), mg/dL	177.0 (119.8)	170.2 (114.5)	171.8 (117.3)
Cholesterol, Mean (SD), mg/dL	160.5 (46.0) ^e^	168.0 (45.3)	171.4 (50.8)
HDL cholesterol, mg/dL	41.6 (14.1)	41.7 (12.9)	41.2 (13.0)
Patient Residence			
Rural	31,967 (14)	124,048 (13)	15,866 (13)
Urban	113,071 (49)	448,385 (48)	54,226 (46)
Unknown	84,172 (37)	365,623 (39)	47,582 (40)
PCP visits in the past five year			
0	7511 (3)	32,228 (3)	4251 (4)
1–9	46,709 (20)	205,534 (22)	27,622 (23)
10–19	57,848 (25)	259,443 (28)	34,360 (29)
20–29	47,808 (21)	191,427 (20)	23,551 (20)
≥30	69,334 (30)	249,424 (27)	27,890 (24)
Homeless in past year	10,950 (5)	44,951 (5)	4936 (4)
Long-Term Care in past two year	3866 (2)	14,828 (2)	2752 (2)

Abbreviation: ACEI = Angiotensin-Converting Enzyme Inhibitor, AMI = Acute Myocardial Infarction, ARBs = Angiotensin II Receptor Blockers, BMI = Body Mass Index, BP = Blood Pressure, COPD = Chronic Obstructive Pulmonary Disease, eGFR = Estimated Glomerular Filtration Rate, GLP1 = Glucagon-Like Peptide-1, HbA1c = Hemoglobin A1C, HDL = High-Density Lipoprotein, LDL = Low-Density Lipoprotein, MRAs = Mineralocorticoid Receptor Antagonists, PCP = Primary Care Physician, PPIs = proton pump inhibitors, SGLT2 = Sodium-Glucose Cotransporter-2. ^a^ Diagnoses assessed before the index date. ^b^ A weighted combined comorbidity score. ^c^ Medication prescription assessed one year before the index date ^d^ Latest examination data assessed within one year before the index date. ^e^ With more than a 10% absolute standardized difference compared to patients with normomagnesemia.

**Table 2:** Baseline Characteristics of Patients with Type 2 Diabetes and Hypomagnesemia Initiated on Prescribed Oral Magnesium, Before and After Propensity Score Matching.

Characteristic, *n* (%)	Before Matching (*n* = 211,128)		After Matching (*n* = 40,766)	
	Prescribed Magnesium		Prescribed Magnesium	
	No	Yes	No	Yes
	(*n* = 190,683)	(*n* = 20,445)	(*n* = 20,383)	(*n* = 20,383)
Age, Mean (SD), y	65.6 (10.9)	66.5 (10.1)	66.6 (10.3)	66.5 (10.1)
Sex				
Male	180,965 (95)	19,372 (95)	19,339 (95)	19,311 (95)
Female	9718 (5)	1073 (5)	1044 (5)	1072 (5)
Diabetes Duration, Mean (SD), y	4.5 (4.5)	5.5 (4.8) ^e^	5.6 (4.8)	5.5 (4.8)
Serum Magnesium, Mean (SD), mg/dL	1.6 (0.2)	1.5 (0.2) ^e^	1.5 (0.2)	1.5 (0.2)
Race				
Hispanic	10,559 (6)	772 (4)	713 (3)	770 (4)
non-Hispanic White	127,468 (67)	14,812 (72) ^e^	14,866 (73)	14,760 (72)
non-Hispanic Black	35,208 (18)	3155 (15)	3059 (15)	3150 (15)
Other/Unknown	17,448 (9)	1706 (8)	1745 (9)	1703 (8)
Marital Status				
Single	18,498 (10)	1701 (8)	1701 (8)	1697 (8)
Divorced/Separated	55,079 (29)	5578 (27)	5509 (27)	5561 (27)
Married	101,041 (53)	11,525 (56)	11,504 (56)	11,492 (56)
Widowed	16,065 (8)	1641 (8)	1669 (8)	1633 (8)
Cormobidities ^a^				
Heart Failure	26,785 (14)	3028 (15)	3074 (15)	3019 (15)
AMI	20,489 (11)	2091 (10)	2079 (10)	2084 (10)
Ischemic stroke	17,691 (9)	1734 (8)	1756 (9)	1730 (8)
Hemorrhagic stroke	1659 (1)	145 (1)	149 (1)	145 (1)
Atrial Fibrillation	23,787 (12)	2888 (14)	2925 (14)	2873 (14)
Hyperlipidemia	152,162 (80)	17,385 (85) ^e^	17,286 (85)	17,327 (85)
Hypertension	169,799 (89)	18,882 (92) ^e^	18,752 (92)	18,823 (92)
Anemia	54,195 (28)	6087 (30)	6069 (30)	6063 (30)
Alcohol abuse	41,360 (22)	4497 (22)	4516 (22)	4477 (22)
Liver Disease	27,556 (14)	3029 (15)	2888 (14)	3023 (15)
Respiratory Failure	8394 (4)	590 (3)	600 (3)	590 (3)
COPD	50,423 (26)	5599 (27)	5539 (27)	5579 (27)
Cancer	62,495 (33)	6359 (31)	6280 (31)	6339 (31)
Neuro Disorders	67,338 (35)	7935 (39)	7833 (38)	7909 (39)
Weight Loss	16,505 (9)	1562 (8)	1603 (8)	1557 (8)
Arthritis	87,972 (46)	10,249 (50)	10,144 (50)	10,208 (50)
Gagne Comorbidity Score ^b^	2.2 (2.5)	2.2 (2.4)	2.2 (2.4)	2.2 (2.4)
Comedications ^c^				
Diabetes Medications				
Insulin	36,989 (19)	4719 (23)	4648 (23)	4713 (23)
Metformin	89,716 (47)	11,753 (57) ^e^	11,704 (57)	11,712 (57)
GLP1	2287 (1)	327 (2)	322 (2)	327 (2)
SGLT2 inhibitors	1504 (1)	207 (1)	194 (1)	206 (1)
Other Diabetes Medication	55,719 (29)	7065 (35) ^e^	7051 (35)	7045 (35)
Other Medications				
Thiazides	53,071 (28)	6531 (32)	6459 (32)	6502 (32)
Loop Diuretics	23,632 (12)	3147 (15)	3174 (16)	3136 (15)
PPIs	68,107 (36)	9151 (45) ^e^	8945 (44)	9100 (45)
Vitamin D	33,566 (18)	4828 (24) ^e^	4796 (24)	4811 (24)
ACEIs	90,817 (48)	10,466 (51)	10,397 (51)	10,433 (51)
ARBs	23,504 (12)	3134 (15)	3125 (15)	3117 (15)
Other anti-hypertension	44,324 (23)	5310 (26)	5231 (26)	5289 (26)
Selected beta blockers	62,014 (33)	7251 (35)	7226 (35)	7231 (35)
Non-selected beta blockers	19,443 (10)	2458 (12)	2429 (12)	2445 (12)
Digoxin or Other Inotropes	7016 (4)	884 (4)	920 (5)	879 (4)
Aspirin	40,048 (21)	4405 (22)	4351 (21)	4386 (22)
Anti-platelet	14,750 (8)	1684 (8)	1642 (8)	1676 (8)
Glucocorticoids	19,131 (10)	1992 (10)	1917 (9)	1985 (10)
Statins	111,767 (59)	13,462 (66) ^e^	13,448 (66)	13,411 (66)
Other non-statin lipid lowering	19,304 (10)	2292 (11)	2267 (11)	2279 (11)
Calcium Channel Blocker	51,103 (27)	6198 (30)	6115 (30)	6172 (30)
MRAs	7251 (4)	1086 (5)	1068 (5)	1081 (5)
Health examination data ^d^				
HbA1c, Mean (SD), %	7.4 (1.8)	7.4 (1.6)	7.3 (1.6)	7.4 (1.6)
BMI, Mean (SD), kg/m^2^	30.7 (6.8)	31.3 (6.7)	31.3 (6.9)	31.3 (6.8)
Systolic BP, Mean (SD), mmHg	134.8 (19.8)	133.4 (18.8)	133.3 (19.1)	133.4 (18.8)
Diastolic BP, Mean (SD), mmHg)	76.0 (11.8)	75.7 (11.3)	75.5 (11.3)	75.7 (11.3)
eGFR, Mean (SD), mL/min/1.73 m^2^	75.5 (24.4)	74.2 (22.2)	74.0 (23.3)	74.2 (22.2)
Serum Vitamin D, Mean (SD), ng/mL				
<20	11,780 (6)	1788 (9) ^e^	1804 (9)	1784 (9)
20–30	17,328 (9)	2499 (12) ^e^	2494 (12)	2492 (12)
30–100	28,700 (15)	4413 (22) ^e^	4453 (22)	4389 (22)
Unknown	132,875 (70)	11,745 (57) ^e^	11,632 (57)	11,718 (57)
Serum Sodium, Mean (SD), mEq/L	137.7 (3.7)	138.1 (3.5) ^e^	138.1 (3.6)	138.1 (3.5)
Serum Potassium, Mean (SD), mEq/L	4.1 (0.5)	4.1 (0.5)	4.1 (0.5)	4.1 (0.5)
Serum Calcium, Mean (SD), mg/dL	9.1 (0.8)	9.2 (0.7) ^e^	9.2 (0.8)	9.2 (0.7)
LDL cholesterol, Mean (SD), mg/dL	87.0 (35.3)	82.2 (34.0) ^e^	81.8 (33.9)	82.2 (34.1)
Triglycerides, Mean (SD), mg/dL	177.6 (120.1)	178.8 (121.3)	177.9 (119.5)	178.8 (121.3)
Cholesterol, Mean (SD), mg/dL	161.2 (46.0)	155.6 (43.7) ^e^	154.9 (41.1)	155.6 (43.7)
HDL cholesterol, mg/dL	41.5 (13.8)	42.3 (15.2)	42.5 (15.0)	42.3 (15.2)
Patient Residence				
Rural	26,004 (14)	3283 (16)	3291 (16)	3264 (16)
Urban	93,229 (49)	10,263 (50)	10,284 (50)	10,232 (50)
Unknown	71,450 (37)	6899 (34)	6808 (33)	6887 (34)
PCP visits in the past five year				
0	6542 (3)	542 (3)	545 (3)	540 (3)
1–9	40,173 (21)	3394 (17) ^e^	3384 (17)	3388 (17)
10–19	48,662 (26)	4946 (24)	5105 (25)	4932 (24)
20–29	39,645 (21)	4481 (22)	4341 (21)	4462 (22)
≥30	55,661 (29)	7082 (35) ^e^	7008 (34)	7061 (35)
Homeless in past year	9112 (5)	807 (4)	831 (4)	806 (4)
Long-Term Care in past two year	3080 (2)	172 (1)	181 (1)	172 (1)

Abbreviation: ACEI = Angiotensin-Converting Enzyme Inhibitor, AMI = Acute Myocardial Infarction, ARBs = Angiotensin II Receptor Blockers, BMI = Body Mass Index, BP = Blood Pressure, COPD = Chronic Obstructive Pulmonary Disease, eGFR = Estimated Glomerular Filtration Rate, GLP1 = Glucagon-Like Peptide-1, HbA1c = Hemoglobin A1C, HDL = High-Density Lipoprotein, LDL = Low-Density Lipoprotein, MRAs = Mineralocorticoid Receptor Antagonists, PCP = Primary Care Physician, PPIs = proton pump inhibitors, SGLT2 = Sodium-Glucose Cotransporter-2. ^a^ Diagnoses assessed before the index date. ^b^ A weighted combined comorbidity score. ^c^ Medication prescription assessed one year before the index date. ^d^ Latest examination data assessed within one year before the index date. ^e^ With more than a 10% absolute standardized difference compared to patients with no prescribed magnesium before propensity score matching

**Table 3:** Outcomes During One Year of Follow-Up for Patients With Type-2 Diabetes and Hypomagnesemia (Top Panel) and Diabetes and Normomagnesemia (Bottom Panel) by Prescribed Magnesium.

	Before Propensity Score Matching			After Propensity Score Matching		
	**Events (%) by prescribed magnesium in patient with hypomagnesemia**		**Adjusted HR ^a^ (95% CI)**	**Events (%) by prescribed magnesium in patient with hypomagnesemia**		**HR ^b^ (95% CI)**
	**No (*n* = 190,683)**	**Yes (*n* = 20,445)**		**No (*n* = 20,383)**	**Yes (*n* = 20,383)**	
MACE	29,846 (15.7%)	2772 (13.6%)	0.93 (0.89–0.96)	3076 (15.1%)	2764 (13.6%)	0.89 (0.84–0.93)
All-cause mortality	14,410 (7.6%)	1267 (6.2%)	0.93 (0.88–0.99)	1452 (7.1%)	1263 (6.2%)	0.86 (0.80–0.93)
		
	**Events (%) by prescribed magnesium in patient with normomagnesemia**		**Adjusted HR ^a^ (95% CI)**	**Events (%) by prescribed magnesium in patient with normomagnesemia**		**HR ^b^ (95% CI)**
	**No (*n* = 887,849)**	**Yes (*n* = 5919)**		**No (*n* = 5919)**	**Yes (*n* = 5919)**	
MACE	115,632 (13.0%)	970 (16.4%)	1.14 (1.07–1.21)	912 (15.4%)	970 (16.4%)	1.07 (0.97–1.17)
All-cause mortality	48,537 (5.5%)	391 (6.6%)	1.11 (1.01–1.23)	389 (6.6%)	391 (6.6%)	1.00 (0.87–1.15)

Abbreviation: CI = Confidence Interval; HR = Hazard Ratio; MACE = major adverse cardiovascular events. ^a^ The adjusted hazard ratios are associated with prescribed oral magnesium when compared with no prescribed oral magnesium, assessed using a multivariable Cox regression model, adjusted for 64 covariates. These include demographic information (age, sex, race, etc.), clinical characteristics (comorbidities, comedications, health examination data, etc.), healthcare utilization metrics, geographic information, and index year. ^b^ The hazard ratios are associated with prescribed oral magnesium when compared with no Prescribed oral magnesium.

## Data Availability

The data that support the findings of this study are derived from the VHA CDW. Restrictions apply to the availability of these data, which were used under the data-user agreement for this study. Data are available to VHA researchers upon adequate approval.

## Supplementary Materials

The following supporting information can be downloaded at: https://www.mdpi.com/article/10.3390/nu17132067/s1, Figure S1: Love Plots Displaying the Absolute Standardized Difference of 64 Baseline Characteristics between Patients with Type-2 Diabetes and (A) Hypomagnesemia, or (B) Normomagnesemia, Who Were and Were Not Initiated on Prescribed Magnesium Before and After Propensity Score Matching; Figure S2: One-Year Kaplan-Meier Curves for Survival by Initiation of Prescribed Magnesium in Propensity Score-Matched Cohorts of Patients with Type 2 Diabetes and (A) Hypomagnesemia, or (B) Normomagnesemia; Figure S3: Hazard Ratios for the Association of Prescribed Magnesium and Time to MACE Within One Year in Subgroup-Specific Propensity-Matched Cohorts of Patients With Type 2 Diabetes and Normomagnesemia; Table S1: Diagnosis Codes; Table S2: Medication List; Table S3: Lab Test List; Table S4: Listing of Continuous Variables with Missing Data; Table S5: Baseline Characteristics of Patients with Type-2 Diabetes and Normomagnesemia Initiated on Prescribed Oral Magnesium, Before and After Propensity Score Matching.

## Abbreviations

The following abbreviations are used in this manuscript:

ACEI	Angiotensin-Converting Enzyme Inhibitor
AMI	Acute Myocardial Infarction
ARBs	Angiotensin II Receptor Blockers
ASD	Absolute Standardized Differences
CDW	Corporate Data Warehouse
CI	Confidence Interval
COPD	Chronic Obstructive Pulmonary Disease
CVD	Cardiovascular Disease
EMR	Electronic Medical Record
GLP1	Glucagon-Like Peptide-1
ICD	International Classification of Diseases
MACE	Major Adverse Cardiovascular Events
MRAs	Mineralocorticoid Receptor Antagonists
PCP	Primary Care Physician
PPIs	Proton Pump Inhibitors
PS	Propensity Scores
SGLT2	Sodium-Glucose Cotransporter-2
VHA	Veterans Health Administration
VISN	Veterans Integrated Services Network
