# Supplementary material for: Serum Magnesium, Prescribed Magnesium Replacement and Cardiovascular Events in Adults with Type 2 Diabetes: A National Cohort Study in U.S. Veterans
Source: Nutrients. 2025 Jun 21;17(13):2067. doi: 10.3390/nu17132067 (PMC12251466; doi:10.3390/nu17132067)
Supplement: Supplementary file 1 [file nutrients-17-02067-s001.zip › nutrients-3689640-supplementary.pdf]

Figure S1: Love Plots Displaying the Absolute Standardized Difference of 64 Baseline Characteristics between Patients with Type-2 Diabetes and A) Hypomagnesemia, or B) Normomagnesemia, Who Were and Were Not Initiated on Prescribed Magnesium Before and After Propensity Score Matching

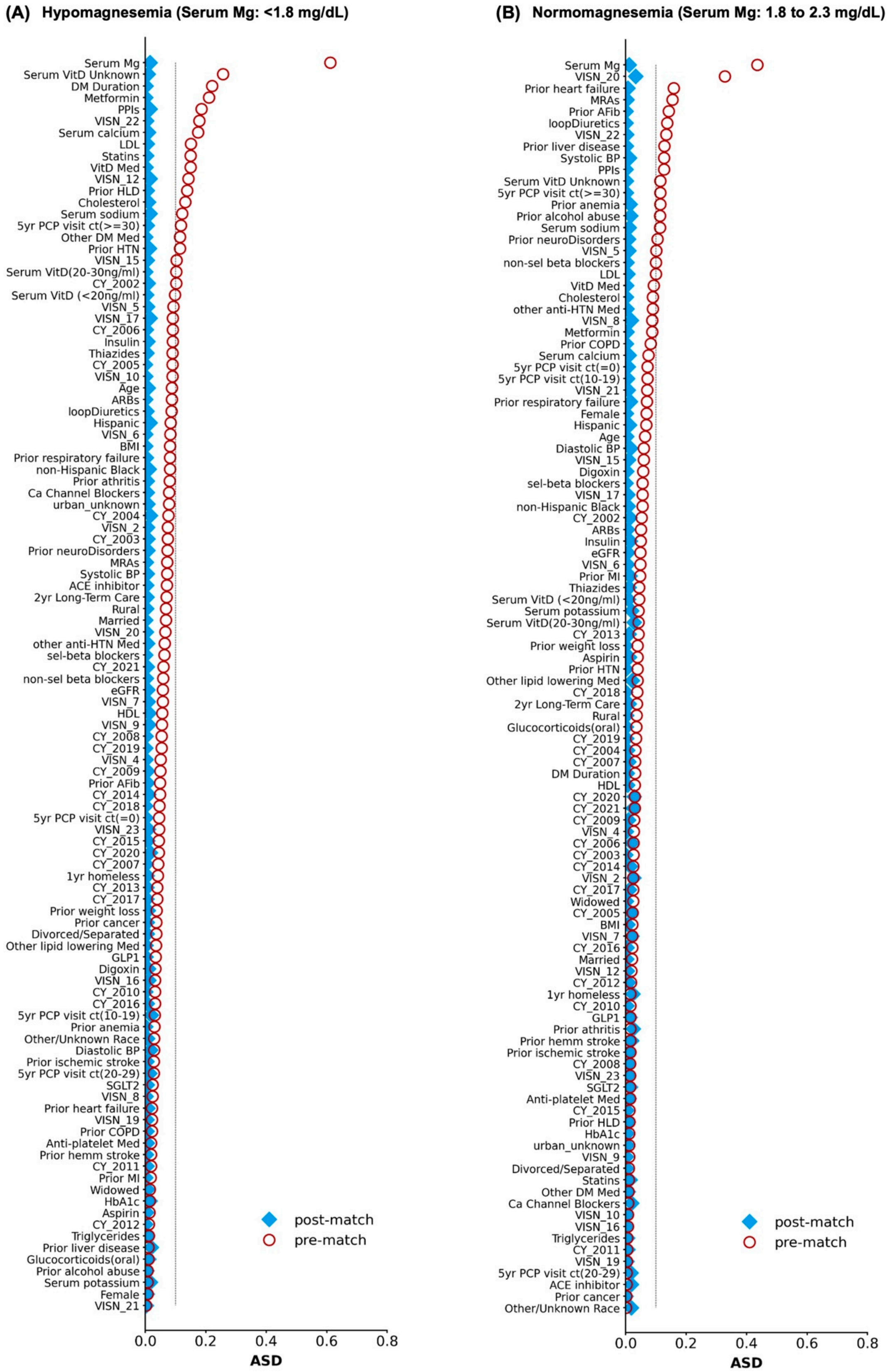

Figure S2: One-Year Kaplan-Meier Curves for Survival by Initiation of Prescribed Magnesium in Propensity Score-Matched Cohorts of Patients with Type 2 Diabetes and A) Hypomagnesemia, or B) Normomagnesemia

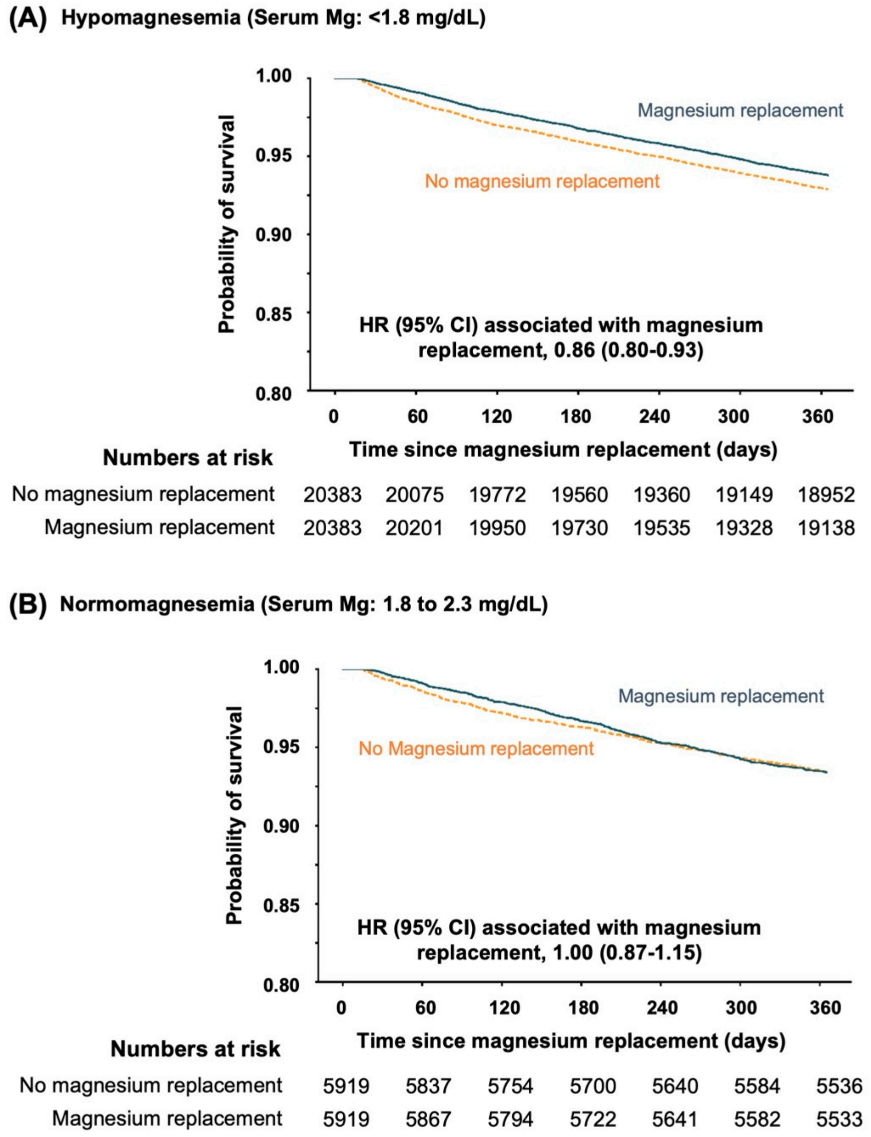

Figure S3. Hazard Ratios for the Association of Prescribed Magnesium and Time to MACE Within One Year in Subgroup-Specific Propensity-Matched Cohorts of Patients With Type 2 Diabetes and Normomagnesemia

Subgroups	No Mg replacement	Mg replacement	Hazard Ratio (95% CI)	P for Interaction
**Age**				0.642
>=65 (n=5874)	531/2937 (18%)	604/2937 (21%)	1.15 (1.02-1.29)	
<65 (n=5964)	334/2982 (11%)	366/2982 (12%)	1.10 (0.95-1.28)	
**Gender**				0.240
Female (n=750)	29/375 (8%)	23/375 (6%)	0.79 (0.46-1.36)	
Male (n=11088)	870/5544 (16%)	947/5544 (17%)	1.10 (1.00-1.20)	
**Race**				0.887
Black (n=2032)	135/1016 (13%)	142/1016 (14%)	1.05 (0.83-1.33)	
non_Black (n=9806)	778/4903 (16%)	828/4903 (17%)	1.07 (0.97-1.18)	
**Prior heart failure**				0.793
Yes (n=2380)	441/1190 (37%)	466/1190 (39%)	1.07 (0.94-1.22)	
No (n=9458)	484/4729 (10%)	504/4729 (11%)	1.04 (0.92-1.18)	
**PPIs**				0.007
Yes (n=4244)	353/2122 (17%)	330/2122 (16%)	0.93 (0.80-1.08)	
No (n=7594)	537/3797 (14%)	640/3797 (17%)	1.21 (1.08-1.36)	
**Metformin use**				0.234
Yes (n=4270)	230/2135 (11%)	250/2135 (12%)	1.09 (0.91-1.30)	
No (n=7568)	593/3784 (16%)	720/3784 (19%)	1.24 (1.11-1.38)	
**Thiazides use**				0.610
Yes (n=3180)	230/1590 (14%)	231/1590 (15%)	1.00 (0.83-1.20)	
No (n=8658)	703/4329 (16%)	739/4329 (17%)	1.06 (0.95-1.17)	
**Loop diuretics use**				0.286
Yes (n=2144)	317/1072 (30%)	331/1072 (31%)	1.06 (0.91-1.24)	
No (n=9690)	545/4845 (11%)	639/4845 (13%)	1.18 (1.05-1.33)	
**Vitamin D use**				0.076
Yes (n=2272)	153/1136 (13%)	147/1136 (13%)	0.96 (0.77-1.20)	
No (n=9560)	694/4780 (15%)	823/4780 (17%)	1.20 (1.09-1.33)	
**Overall**	912/5919 (15%)	970/5919 (16%)	1.07 (0.97-1.17)	

MACE within 1 year Event/Total (%)

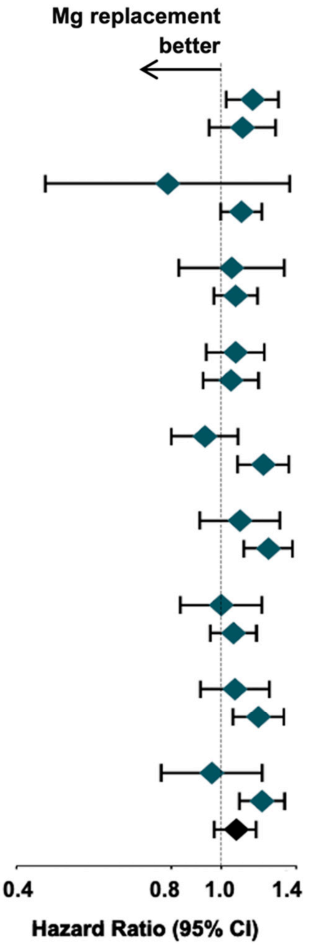

Table S1: Diagnosis Codes

Condition	ICD-9CM	ICD-10CM
Type 2 Diabetes	250.X,357.2X,362.0X,366.41,648.0X	E11.x, O24.1x
AMI	410.x, 412.x	I21.x, I22.x, I25.2x
Heart Failure	428.x,398.91,402.01,402.11, 402.91,404.01,404.03,404.11, 404.13,404.91,404.98	I50.x, I11.0x, I13.0x, I13.2x
Ischemic stroke	436.x, 438.x,433.01,433.11, 433.21,433.31,433.81,433.91, 434.01,434.11,434.91	G46.x, I63.x, I69.3x, I69.8x, I69.9x
Hemorrhagic stroke	430.x, 431.x, 432.x	I60, I61, I62, I69.0, I69.1, I69.2
Atrial Fibrillation	427.31	I48.0x, I48.1x, I48.2x, I48.91
Hyperlipidemia	272.0x,272.1x,272.2x,272.3x,272.4x	E78.0x, E78.1x, E78.2x, E78.3x, E78.4x, E78.5x
Hypertension	401.x	I10.x, I16.x
Alcohol abuse	303.x,305.0x	F10.x
Anemia	280.x-285.x	D50.x-D53.x, D55.x-D64.x
Arthritis	715.x, 714.x,716.2x,716.3x, 716.5x,716.6x	M05.x-M08.x, M10.x, M13.x, M1A.x, M15.x-M19.x
Cancer	140.x -208.x, 230.x-239.x	C00.x-D09.x D37.x-D49.x
COPD	491.x, 492.x, 496.x	J41.x, J42.x, J43.x, J44.x
Liver Disease	570.x-573.x	B15.x-B19.x, K70.x-K77.x
Neuro Disorders	320.x-327.x, 330.x-337.x, 339.x-341.x, 345.x-349.x	G00.x-G14.x, G21.x-G26.x,G30.x-G32.x, G35.x-G37.x, G40, G43, G44, G47
Respiratory Failure	799.1,518.51,518.53,518.81,518.84	J96, J80, R09.2, J95.82
Weight Loss	260.x-263.x,799.4,783.2,783.7	E40.x-E46.x, R64.x, R63.4x

Table S2: Medication List

Medication Group	Drug Name (generic)
Diabetes Medications	
Insulin	Insulin
Metformin	Metformin
GLP-1 receptor agonists	Albiglutide, Dulaglutide, Liraglutide, Lixisenatide, Semaglutide
SGLT-2 Inhibitors	Canaglifozin, Dapagliflozin, Empagliflozin
Others	Thiazolidinediones: Pioglitazone, Rosiglitazone, Troglitazone
Cardiovascular Medications	
ACEIs	Benazepril, Captopril, Enalapril, Enalaprilat, Fosinopril, Lisinopril, Moexipril, Perindopril, Quinapril, Ramipril, Trandolapril
ARBs	Azilsartan,Candesartan,Eprosartan,Irbesartan,Losartan, Olmesartan,Telmisartan,Valsartan
Other anti-hypertensive medications	Aliskiren, Amiloride, Chlorpromazine, Chlorthalidone, Clonidine, Doxazosin, Eplerenone, Hydralazine, Methyldopa, Methyldopate, Nitroprusside, Phenoxybenzamine, Prazosin, Spironolactone, Terazosin, Tolazoline, Triamterene
Anti-platelets	Abciximab, Cangrelor, Cilostazol, Clopidogrel, Dipyridamole, Eptifibatide, Prasugrel, Ticagrelor, Ticlopidine, Tirofiban, Asprin
Selective beta blockers	Acebutolol, Atenolol, Betaxolol, Bisoprolol, Esmolol, Metoprolol, Nebivolol
Non-selective beta blockers	Carteolol, Carvedilol, Labetalol, Levobunolol, Metipranolol, Nadolol, Penbutolol, Pindolol, Propranolol, Timolol
Calcium Channel Blockers	Amlodipine, Clevidipine, Diltiazem, Felodipine, Isradipine, Levamlodipine, Nicardipine, Nifedipine, Nimodipine, Verapamil
Digoxin and other Inotropes	Amrinone Inj, Digoxin, Dobutamine Inj, Dopamine Inj, Disopyramide, Epinephrine, Ethylnorepinephrine Inj, Flecainide, Inamrinone Inj, Isoproterenol Inj, Milrinone,Norepinephrine Inj, Procainamide, Quinidine, Sunitinib, Theophylline
Loop Diuretics	Bumetanide, Ethacrynic Acid, Furosemide
MRAs	Eplerenone, Finerenone, Spironolactone
Other non-statin lipid lowering	Alirocumab, Cholestyramine, Clofibrate, Colesevelam, Colestipol, Evolocumab, Ezetimibe, Fenofibrate, Fenofibric Acid, Gemfibrozil, Inclisiran, Lomitapide, Niacin
Statins	Atorvastatin, Cerivastatin, Fluvastatin, Lovastatin, Pitavastatin, Pravastatin, Rosuvastatin, Simvastatin
Thiazides	Chlorothiazide, Hydrochlorothiazide, Indapamide, Methyclothiazide, Metolazone
Select Non-Cardiovascular Medications	
Vitamin D	Ergocalciferol (Vitamin D2), Cholecalciferol (Vitamin D3)
Glucocorticoids	Cortisone, Dexamethasone, Fludrocortisone, Hydrocortisone, Methylprednisolone, Prednisolone, Prednisone
PPIs	Dexlansoprazole, Esomeprazole, Omeprazole, Panteprazole, Rabeprazole

Table S3: Lab Test List

Lab	LOINC Code
**Creatinine (mg/dL)***	77140-2, 14682-9, 2160-0, 40248-7, 40264-4
**HbA1c (%)**	17855-8, 17856-6, 41995-2, 43150-2, 4548-4, 4549-2, 54039-3, 55454-3, 59261-8, 62388-4, 62853-7, 62854-5, 67761-7, 71875-9, 74246-0, 75862-3, 86910-7
**Cholesterol (mg/dL)**	2093-3, 48620-9, 35200-5, 14647-2
**HDL cholesterol (mg/dL)**	2085-9, 18263-4, 35197-3, 27340-9, 14646-4, 96596-2, 2086-7, 49130-8, 12771-2, 12772-0
**LDL cholesterol (mg/dL)**	12773-8, 49132-4, 35198-1, 14155-6, 96597-0, 2089-1, 2090-9, 13457-7, 96259-7, 18262-6, 55440-2, 18261-8, 22748-8, 39469-2, 96258-9, 69419-0
**Triglycerides (mg/dL)**	96598-8, 14927-8, 3043-7, 3049-4, 2571-8, 12951-0, 35217-9, 30570-6, 28554-4, 70218-3
**Serum Calcium (mg/dl)**	17861-6, 2000-8, 49765-1, 17864-0
**Serum Magnesium (mg/dl)**	19123-9, 21377-7, 2593-2, 2600-5, 2601-3, 32698-3
**Serum Potassium (mEq/L)**	22760-3, 42569-4, 2823-3, 77142-8
**Serum Sodium (mEq/L)**	42570-2, 2951-2, 77139-4, 44783-9
**Serum Vitamin D (ng/mL)**	1989-3, 62292-8, 35365-6, 1679-0, 62290-2, 62291-0, 49054-0, 2439-8, 14635-7
* eGFR (mL/min/1.73m2) were calculated using serum creatinine based on Chronic Kidney Disease Epidemiology Collaboration (CKD-EPI) equations	

Table S4: Listing of Continuous Variables with Missing Data

	Hypomagnesemia (Serum Magnesium: <1.8 mg/dL)		Normomagnesemia (Serum Magnesium: 1.8 to 2.3 mg/dL)	
	**Prescribed Magnesium**		**Prescribed Magnesium**	
	**No**	**Yes**	**No**	**Yes**
**Variables[a], N (%)**	**(n= 190,683)**	**(n= 20,445)**	**(n= 887,849)**	**(n= 5,919)**
HbA1c	22954 (12%)	1236 (6%)	116207 (13%)	705 (12%)
BMI	11165 (6%)	1036 (5%)	52955 (6%)	254 (4%)
Serum Sodium	3556 (2%)	301 (1%)	17703 (2%)	128 (2%)
Serum Potassium	2949 (2%)	256 (1%)	16876 (2%)	198 (3%)
Serum Calcium	10683 (6%)	1440 (7%)	53396 (6%)	401 (7%)
Systolic BP	5132 (3%)	552 (3%)	25172 (3%)	104 (2%)
Diastolic BP	5132 (3%)	552 (3%)	25172 (3%)	104 (2%)
LDL cholesterol	31814 (17%)	2231 (11%)	131389 (15%)	894 (15%)
Triglycerides	29631 (16%)	2274 (11%)	125421 (14%)	821 (14%)
Cholesterol	28370 (15%)	1907 (9%)	121090 (14%)	778 (13%)
HDL cholesterol	26095 (14%)	1837 (9%)	111487 (13%)	697 (12%)
eGFR	5150 (3%)	537 (3%)	32938 (4%)	178 (3%)

[a]Lab test and vital sign data assessed within one year before the index date; if there is no data available, then consider it as missing. Missing data were imputed by single imputation using a fitted general linear model on age, sex, race and ethnicity

Table S5. Baseline Characteristics of Patients with Type-2 Diabetes and Normomagnesemia Initiated on Prescribed Oral Magnesium, Before and After Propensity Score Matching

	Before matching (n=893,768)		After matching (n=11,838)	
	Prescribed Magnesium		Prescribed Magnesium	
	No	Yes	No	Yes
**Characteristic, N (%)**	(n= 887,849)	(n= 5,919)	(n= 5,919)	(n= 5,919)
**Age, Mean (SD), y**	65.4 (11.8)	64.7 (11.3)	64.7 (11.7)	64.7 (11.3)
**Gender**				
Male	845672 (95)	5544 (94)	5542 (94)	5544 (94)
Female	42177 (5)	375 (6)	377 (6)	375 (6)
**Diabetes Duration, Mean (SD), y**	3.6 (4.0)	3.7 (4.2)	3.7 (4.1)	3.7 (4.2)
**Serum Magnesium, Mean (SD), mg/dL**	2.03 (0.15)	1.96 (0.16)[e]	1.96 (0.14)	1.96 (0.16)
**Race**				
Hispanic	52710 (6)	262 (4)	277 (5)	262 (4)
non-Hispanic White	574711 (65)	4046 (68)	4016 (68)	4046 (68)
non-Hispanic Black	171271 (19)	1016 (17)	998 (17)	1016 (17)
Other/Unknown	89157 (10)	595 (10)	628 (11)	595 (10)
**Marital Status**				
Single	86810 (10)	532 (9)	530 (9)	532 (9)
Divorced/Separated	243638 (27)	1652 (28)	1660 (28)	1652 (28)
Married	480844 (54)	3264 (55)	3255 (55)	3264 (55)
Widowed	76557 (9)	471 (8)	474 (8)	471 (8)
**Cormobidities[a]**				
Heart Failure	125568 (14)	1190 (20)[e]	1173 (20)	1190 (20)
AMI	95152 (11)	724 (12)	697 (12)	724 (12)
Ischemic stroke	79737 (9)	507 (9)	524 (9)	507 (9)
Hemorrhagic stroke	7283 (1)	41 (1)	32 (1)	41 (1)
Atrial Fibrillation	105248 (12)	996 (17)[e]	998 (17)	996 (17)
Hyperlipidemia	704870 (79)	4671 (79)	4653 (79)	4671 (79)
Hypertension	758041 (85)	5133 (87)	5131 (87)	5133 (87)
Anemia	203332 (23)	1649 (28)[e]	1612 (27)	1649 (28)
Alcohol abuse	160005 (18)	1337 (23)[e]	1378 (23)	1337 (23)
Liver Disease	93063 (10)	871 (15)[e]	874 (15)	871 (15)
Respiratory Failure	30089 (3)	282 (5)	263 (4)	282 (5)
COPD	226104 (25)	1723 (29)	1729 (29)	1723 (29)
Cancer	269316 (30)	1799 (30)	1797 (30)	1799 (30)
Neuro Disorders	312089 (35)	2380 (40)[e]	2354 (40)	2380 (40)
Weight Loss	62253 (7)	476 (8)	476 (8)	476 (8)
Arthritis	411169 (46)	2785 (47)	2712 (46)	2785 (47)
Gagne Comorbidity Score[b]	1.9 (2.4)	2.3 (2.5)[e]	2.3 (2.6)	2.3 (2.5)
**Comedications[c]**				
Diabetes Medications				
Insulin	108472 (12)	822 (14)[e]	853 (14)	822 (14)
Metformin	283582 (32)	2135 (36)	2146 (36)	2135 (36)
GLP1	5780 (1)	46 (1)	39 (1)	46 (1)
SGLT2 inhibitors	8522 (1)	65 (1)	75 (1)	65 (1)
Other Diabetes Medication	185068 (21)	1213 (20)	1233 (21)	1213 (20)

Other Medications				
Thiazides	221249 (25)	1590 (27)	1561 (26)	1590 (27)
Loop Diuretics	116892 (13)	1073 (18)[e]	1072 (18)	1073 (18)
PPIs	265782 (30)	2122 (36)[e]	2120 (36)	2122 (36)
Vitamin D	139702 (16)	1139 (19)	1133 (19)	1139 (19)
ACEIs	375259 (42)	2494 (42)	2547 (43)	2494 (42)
ARBs	97089 (11)	743 (13)	732 (12)	743 (13)
Other anti-hypertension	195342 (22)	1529 (26)	1520 (26)	1529 (26)
Selected beta blockers	257551 (29)	1868 (32)	1866 (32)	1868 (32)
Non-selected beta blockers	81962 (9)	730 (12)[e]	722 (12)	730 (12)
Digoxin or Other Inotropes	36528 (4)	316 (5)	313 (5)	316 (5)
Aspirin	180812 (20)	1299 (22)	1328 (22)	1299 (22)
Anti-platelet	65983 (7)	419 (7)	403 (7)	419 (7)
Glucocorticoids	86120 (10)	637 (11)	634 (11)	637 (11)
Statins	495418 (56)	3274 (55)	3314 (56)	3274 (55)
Other non-statin lipid lowering	85265 (10)	503 (8)	469 (8)	503 (8)
Calcium Channel Blocker	226774 (26)	1532 (26)	1483 (25)	1532 (26)
MRAs	27328 (3)	376 (6)[e]	370 (6)	376 (6)
**Health examination data[d]**				
HbA1c, Mean (SD), %	7.0 (1.6)	7.1 (1.7)	7.1 (1.7)	7.1 (1.7)
BMI, Mean (SD), kg/m2	30.8 (6.6)	31.0 (6.9)	30.9 (6.7)	31.0 (6.9)
Systolic BP, Mean (SD), mmHg	134.8 (19.0)	132.3 (19.0)[e]	132.5 (19.0)	132.3 (19.0)
Diastolic BP, Mean (SD), mmHg)	76.5 (11.7)	75.8 (11.7)	76.0 (11.8)	75.8 (11.7)
eGFR, Mean (SD), mL/min/1.73m2	75.0 (23.3)	73.8 (24.7)	73.9 (24.1)	73.8 (24.7)
Serum Vitamin D, Mean (SD), ng/mL				
<20	54513 (6)	428 (7)	414 (7)	428 (7)
20-30	84101 (9)	636 (11)	687 (12)	636 (11)
30-100	127068 (14)	1025 (17)	993 (17)	1025 (17)
Unknown	622167 (70)	3830 (65)[e]	*3825 (65)*	3830 (65)
Serum Sodium, Mean (SD), mEq/L	138.4 (3.4)	138.0 (3.8)[e]	138.0 (3.5)	138.0 (3.8)
Serum Potassium, Mean (SD), mEq/L	4.2 (0.5)	4.2 (0.6)	4.2 (0.5)	4.2 (0.6)
Serum Calcium, Mean (SD), mg/dL	9.2 (0.6)	9.2 (0.8)	9.2 (0.7)	9.2 (0.8)
LDL cholesterol, Mean (SD), mg/dL	95.1 (38.8)	91.0 (35.5)[e]	91.2 (35.2)	91.0 (35.5)
Triglycerides, Mean (SD), mg/dL	170.6 (114.7)	169.4 (117.7)	168.4 (114.8)	169.4 (117.7)
Cholesterol, Mean (SD), mg/dL	168.3 (45.2)	164.0 (46.5)	163.8 (43.3)	164.0 (46.5)
HDL cholesterol, mg/dL	41.7 (12.8)	42.2 (13.8)	42.3 (13.6)	42.2 (13.8)
**Patient Residence**				
Rural	116516 (13)	850 (14)	856 (14)	850 (14)
Urban	421988 (48)	2772 (47)	2751 (46)	2772 (47)
Unknown	349345 (39)	2297 (39)	*2312 (39)*	2297 (39)
**PCP visits in the past five year**				
0	30911 (3)	291 (5)	281 (5)	291 (5)
1-9	196916 (22)	1116 (19)	1147 (19)	1116 (19)
10-19	247240 (28)	1462 (25)	1477 (25)	1462 (25)
20-29	181158 (20)	1200 (20)	1159 (20)	1200 (20)
>=30	231624 (26)	1850 (31)[e]	1855 (31)	1850 (31)
**Homeless in past year**	42453 (5)	303 (5)	334 (6)	303 (5)
**Long-Term Care in past two year**	13077 (1)	63 (1)	70 (1)	63 (1)

[a]Diagnoses assessed before the index date.
[b]A weighted combined comorbidity score.
[c]Medication prescription assessed one year before the index date
[d]Latest examination data assessed within one year before the index date
[e]With more than a 10% absolute standardized difference compared to patients with no prescribed magnesium before propensity score matching
